# Effect of a phytogenic feed additive on performance, ovarian morphology, serum lipid parameters and egg sensory quality in laying hen

**Published:** 2014

**Authors:** Ali Asghar Saki, Hassan Aliarabi, Sayed Ali Hosseini Siyar, Jalal Salari, Mahdi Hashemi

**Affiliations:** 1 *Department of Animal Science, Faculty of Agriculture, Bu-Ali Sina University, Hamedan, Iran; *; 2 * Department of Analytic Chemistry, Faculty of Chemistry, Bu-Ali Sina University, Hamedan, Iran.*

**Keywords:** Herbal medicine, Laying performance, Ovary follicle, Sensory evaluation, Trimethylaminuria

## Abstract

This present study was conducted to evaluate the effects of dietary inclusion of 4, 8 and 12 g kg^-1^ phytogenic feed additives mixture on performance, egg quality, ovary parameters, serum biochemical parameters and yolk trimethylamine level in laying hens. The results of experiment have shown that egg weight was increased by supplementation of 12 g kg^-1^ feed additive whereas egg production, feed intake and feed conversion ratio (FCR) were not significantly affected. There were no significant differences in egg quality parameters by supplementation of phytogenic feed additive, whereas yolk trimethylamine level was decreased as the feed additive level increased. The sensory evaluation parameters did not differ significantly. No significant differences were found in serum cholesterol and triglyceride levels between the treatments but low- and high-density lipoprotein were significantly increased. Number of small follicles and ovary weight were significantly increased by supplementation of 12 g kg^-1 ^feed additive. Overall, dietary supplementation of polyherbal additive increased egg weigh, improved ovary characteristics and declined yolk trimethylamine level.

## Introduction

Phytogenic feed additives are materials derived from plants to improve animal performance. These products potentially supply antioxidative effects, increase palatability, improve gut functions, and promote growth and hypo-lipidemic effects.^1^^,^^2^

Several reports have described the effects of various phytogenic additives on poultry production. Frequent studies have explained the property of garlic (*Allium sativa*) on increased egg production, reduced cholesterol content of serum and yolk and improved immune response in layers.^1^^,^^3^^,^^4^ Aydin *et al*. have shown that black cumin would positively influence the performance, egg quality and decrease the concentration of cholesterol in the egg yolk.^5^ Cabuk *et al*. have shown that only feed conversation ratio (FCR) improved by supplementation of an essential oil mixture (Oregano, laurel leaf, sage leaf, myrtle leaf, fennel seeds and citrus peel and 24 mg essential oils per kg feed).^6^ It is reported that average egg production in broiler breeder was improved by polyherbal essential oils feed additive.^7^ Mohammed and Abbas noted that the dietary fennel seed improved weight gain, feed efficiency and blood characteristic in broilers.^8^ Frankic *et al*. noted that marigold *(Calendula* *officinalis)* extracts protected the organism against DNA damage.^9^

The stimulatory effect of phytogenic additives on feed intake is due to the maintained improvement in palatability of the diet resulting from the enhanced flavor and odor and thus enhancing performance.^10^^,^^11^

In laying quails, dietary garlic powder supplementation caused differences in feed consumption, feed efficiency and egg production as averaged over 12 weeks.^12^ However, in another study, no effects were observed by using oregano in laying quails.^13^ In addition, no responses were found in egg production, feed consumption and feed efficiency by adding 0, 5 and 10 g kg^-1^ garlic powder in laying hens diet.^3^ These results could be due to differences in origin, preparation methods, stability of effective substrates, and reaction mechanism of phytogenic feed additives. 

It would be favorable for human health to supply the low cholesterol poultry products. Some herbal additives have potential hypolipidemic and hypocholesterolemic effects. Serum and egg yolk cholesterol concentrations were decreased linearly by increasing levels of garlic from 20 to 100 g kg^-1^ and 20 or 30 g kg^-1 ^black cumin.^1^^,^^5^ No significant effects were observed byfeeding1 and 2 g kg^-1^ dietary medicinal herbs on plasma levels of cholesterol, triglyceride, high-density lipoprotein (HDL) and low-density lipoprotein (LDL).^14^

Trimethylamine (TMA), the compound responsible for the fishy smell, is produced by bacterial fermentation in the lower gut.^15^ In humans, trimethylaminuria is a metabolic disorder due to accumulation of TMA that dietary aspects can influence TMA metabolism in humans.^16^ One of the ways to mitigate this syndrome is preventing use of dietary sources of TMA such as egg yolk.^17^ It is suggested that adding the antibiotics agents to the laying diet could reduce the incidence of fishy egg.^18^ Some phytogenic feed additives have antibiotic properties. It is demonstrated that garlic, thyme, milfoil, fennel seed have remarkable antibacterial effects,^19^^,^^20^ that may decrease TMA concentration in yolk.

Thus, the present study was conducted to characterize the effect of phytogenic feed additive mixture on performance, egg quality, and lipid related serum metabolite, and egg-yolk trimethylamine of laying hens.

## Materials and Methods

**Birds and experimental design. **Ninety-six, 80 week-old white Leghorn laying hens in second production cycle were distributed to 32 cages in a completely randomized design (CRD) for six weeks. Hens were randomized into four experimental groups with four replicates including two cages and each cage contained three birds. The hens were maintained under a 16 hr light-8 hr dark schedule. Water was *ad libitum *and feed were offered 110 g per each bird during the study. Phytogenic feed additive in this study was a commercial product (Shadzi Pasargad Co., Shiraz, Iran) that contained a homogenate mixture of raw herbal powders including garlic (*Allium sativa*), marigold (*Calendula officinalis*), fennel seeds (*Foeniculum vulgare*) and thyme (*thymus vulgaris*).

The basal diet without phytogenic feed additive was served as control, and increasing levels of phytogenic feed additive were supplemented to basal diet in three levels 4, 8 and 12 g kg^-1 ^as experimental groups (Table 1).

**Performance and feedingof laying hen. **Feed consumption was recorded on a replicate basis at weekly intervals. Feed conversion was calculated as the ratio of g feed consumed per g egg weight produced. The eggs were collected daily and egg production, egg weights and egg mass were recorded.

**Egg quality analysis. **All eggs produced at the end of the experiment were collected in two consecutive days and egg quality parameters (albumen weight, albumen height, Haugh unit (HU), shell weight, eggshell thickness, yolk weight, yolk diameter, yolk index and yolk color) were measured. Yolk index was calculated as the ratio of yolk height to yolk width. Yolk color was scored using a colori-metric fan (Roch). Haugh Unit index was also calculated.^21^

**Trimethylamine concentration in egg yolk. **Three yolks of each replicate (total 12 yolk of each treatment) were mixed and a two grams sample was taken to determine TMA concentration and preparation according to Zhang *et al*. method.^22^ TMA was determined using a gas chromatograph (Model GC1014B; Shimazo Corporation, Kyoto, Japan) equipped with an auto sampler, flame ionization detector (FID, Shimazo Corporation, Kyoto, Japan), and fused-silica capillary column (Model CBP1-M25-025; Shimazo Corporation, Kyoto, Japan). From samples, 0.2 μL was injected into the column with nitrogen as a gas carrier (flow rate of 1 mL min^-1^). Oven temperature was set at an initial temperature of 110˚C for 1 min, progressively increased at 15 ˚C per min to 190 ˚C, held for 5 min, then increased at 5 ˚C per min to 230 ˚C and held for 5 min. Inlet and detector temperatures were 250 ˚C. Peak areas and percentages of TMA was calculated.

**Table 1:** Composition ingredient and nutrient of experimental diets including phytogenic feed additive (PFA).

	**Level of PFA (g kg** ^-1^ **)**			
	**Control**	**4**	**8**	**12**
***Ingredient *** ***(g kg*** ^-1^ ***)***				
Corn, grain	489.60	481.50	473.20	465.00
Soybean meal	228.70	230.40	232.00	233.60
Triticale	70.00	70.00	70.00	70.00
Wheat	100.00	100.00	100.00	100.00
Soybean oil	10.20	12.60	15.30	17.90
Phytogenic feed additive	0.00	4.00	8.00	12.00
Di-calcium Phosphate	14.40	14.40	14.40	14.40
Oyster shell	77.20	77.20	77.20	77.20
Salt	4.00	4.00	4.00	4.00
Vitamin permix†	2.50	2.50	2.50	2.50
Mineral premix‡	2.50	2.50	2.50	2.50
DL-Metionine	0.90	0.90	0.90	0.90
***Nutrient (g kg*** ^-1^ ***)***				
Crude protein	165.00	165.00	165.00	165.00
Calcium	33.50	33.50	33.50	33.50
Available phosphorus	4.00	4.00	4.00	4.00
Sodium	1.80	1.80	1.80	1.80
Lysine	8.70	8.70	8.70	8.70
Metionine	3.50	3.50	3.50	3.50
Metionine + cysteine	6.40	6.40	6.40	6.40
Metabolizable energy (MJ kg^-1^)	11.30	11.30	11.30	11.30

† Vitamin premix provided the following per kg of diet: Vitamin A 7,718 IU; Cholecalciferol 2,200 IU; Vitamin E 10 IU; B_12_ 11 mg; Choline 379 mg; Ribofelavin 5.0 mg; Niacin 33 mg; Biotin 0.06; Pyridoxine 0.9; Ethoxyquin 28 mg.

‡ Mineral premix provided following per kg of diet: Mn 55 mg; Zn 40 mg; Fe 28 mg; Cu 7 mg; I 1 mg; Se 0.2 mg.

**Sensory evaluation of eggs. **The sensory evaluation study involved 20 untrained panelists but usual consumers of eggs, who were asked to evaluate eggs according to Hammershoj and Steenfeldt with some modifications.^23^ Eggs were collected three days before the tests and stored at 5 ˚C. Five shell eggs per 750 mL of water were heated at 97 ˚C for 15 min, transferred to cold water for 10 min, peeled, divided into halves, kept in 100-mL closed plastic containers for 30 min at 20 ˚C, and served four eggs to each panelist as coded samples. It was asked to evaluate peeled eggs by cutting each egg in half and evaluating egg aroma, egg flavor, presence of any off-flavor, and overall egg acceptability. These descriptors were quantified by a 9-point hedonic scale (1 = dislike extremely; 9 = like extremely), except off-flavor property which was described by 9-point scale (1 = weak; 9 = strong).^24^

**Biochemical parameters of serum. **Blood samples (3 mL) of eight layers in each treatment, were collected from the brachial wing vein using sterilized syringes and needles at the end of feeding period. After 1 hr standing at room temperature, serum was removed by centrifuge at 1500 *g* for 10 min. Serum samples were stored at –20 ˚C for determination of serum parameters. Serum concentrations of triglyceride, cholesterol, LDL and HDL were measured using commercial kit (Pars Azmoon, Tehran, Iran) and calorimetric method using spectrophotometer.

**Physiological parameters of ovary. **At the end of the experimental period, four layer hens from each treatment were randomly chosen and the ovary was removed and weighed. The number of normal large yellow follicles (>10 mm diameter) and the number of small yellow and white follicles (1 to 10 mm diameter) were recorded.^25^

**Statistical analysis. **The data were analyzed by GLM Procedure of SAS (Version 9.1; SAS Institute, Carry, USA). Comparisons between mean values were done by Duncan’s Multiple Range Test method. Data of sensory evaluation were analyzed by nonparametric procedure. 

## Results

**Laying hen performance. **Phytogenic feed additive supplementation did not affect the laying hen performance (*p *> 0.05). However, egg weight significantly improved by 12 g kg^-1 ^feed additives supplementation (*p *< 0.05). The hens of all treatments had the similar hen-day egg production, egg mass, feed intake and FCR values registered (Table 2).

**Table 2:** Effects of dietary phytogenic feed additive (PFA) levels on laying hens performance.

**Level of PFA (g kg** ^-1^ **)**	**Egg production** **(%)**	**Egg weight (g)**	**Egg mass ** **(g per hen per day)**	**Feed intake** **(g per hen per day)**	**Feed conversation ratio**
**Control**	66.07	61.66^b^	40.94	89.53	2.23
**4**	67.38	64.67^ab^	42.94	98.08	2.25
**8**	69.17	63.79^ab^	45.03	97.79	2.19
**12**	67.88	65.01^a^	43.65	93.83	2.21
***p*** **-value**	0.38	0.04	0.18	0.22	0.42
**SEM**	2.27	1.02	1.33	3.09	0.04

a,b Mean values with different letters in the same column are significantly different (*p *< 0.05).

**Egg quality analysis and egg yolk TMA. **Egg quality parameters and yolk characteristics are presented in Tables 3 and 4, respectively. Albumen height, Haugh unit and shell thickness were not significantly affected by dietary treatments within the six weeks period. No significant differences were recognized in yolk weight, diameter, height, yolk index and color by phytogenic feed additive supplementation (*p *> 0.05). However, TMA was linearly decreased by supplementation of herbal feed additive mixture. All experimental treatments were significantly lower in this respect (*p *< 0.05) in comparison to control. 

**Sensory evaluation. **The results of sensory aspect of eggs at the end of experiment were shown in Table 5. The results for the inclusion of the phytoherbal additive illustrated that no significant differences were observed in egg aroma and flavor between treatment groups (*p *> 0.05). None of the treatments had an off-flavor such as fishy taste in the eggs. All of the eggs were acceptable by panelists.

**Serum Biochemistry. **Serum metabolites including triglyceride, cholesterol, LDL and HDL were presented in Table 6. The serum triglyceride and cholesterol were not affected significantly by inclusion of different levels of phytogenic feed additives in layer diets (*p *> 0.05). However, serum HDL and LDL were increased by inclusion of phytogenic feed additives (*p *< 0.05).

**Reproductive morphology parameters. **The ovary weight was significantly highest with 12 g kg^-1 ^supplementation of herbal feed additive mixture (*p* < 0.05). The number of small follicles (between 1 to 10 mm diameters) was increased by herbal feed additive mixture (*p *< 0.05). In contrast, no reflection was found on large follicles (larger than 10 mm) in this respect, (Table 7).

**Table 3:** Effects of dietary phytogenic feed additive (PFA) levels on laying hens performance.

**Level of PFA (g kg** ^-1^ **)**	**Albumin Weight (g)**	**Albumin Height (mm)**	**Haugh unit**	**Shell thickness (mm)**	**Sell Weight (g)**
**Control**	43.89	6.67	79.25	0.03	5.26
**4**	42.78	7.01	82.10	0.03	5.13
**8**	41.19	6.03	73.66	0.03	5.38
**12**	41.37	6.24	75.69	0.03	5.28
***p*** **-value**	0.53	0.20	0.14	0.17	0.62
**SEM**	1.70	0.34	2.58	0.002	0.12

No significant differences were detected among the treatments.

**Table 4:** Effects of dietary phytogenic feed additive (PFA) levels on laying hens yolk parameters and yolk trimethylamine

**Level of PFA (g kg** ^-1^ **)**	**Yolk Weight** **(g)**	**Yolk Diameter (mm)**	**Yolk Height (mm)**	**Yolk index**	**Yolk color**	**Trimethylamine (µg g** ^-1^ **)**
**Control**	17.70	44.42	17.00	0.38	6.64	0.735^a^
**4**	18.56	43.10	17.61	0.41	7.07	0.715^ab^
**8**	18.73	47.45	17.01	0.37	7.33	0.657^bc^
**12**	18.29	43.16	17.64	0.41	7.20	0.602^c^
***p*** **-value**	0.88	0.41	0.65	0.24	0.40	0.016
**SEM**	0.84	2.38	0.48	0.15	0.35	0.017

No significant differences were detecet among the treatments.

**Table 5.** Effects of dietary phytogenic feed additive (PFA) levels on sensory evaluation (1 to 9 scale).

**Level of PFA** **(g kg** ^-1^ **)**	**Aroma**	**Flavor**	**Off-flavor**	**Overall acceptability**
**Control**	4.89	6.37	2.39	6.83
**4**	5.60	6.00	2.43	6.57
**8**	5.65	6.93	2.26	7.70
**12**	5.05	6.12	2.35	6.76
***p*** **-value**	0.34	0.57	0.90	0.50
**SEM**	0.38	0.56	0.42	0.57

**Table 6:** Effects of dietary phytogenic feed additive (PFA) levels on triglyceride, cholesterol, High- and low- density lipoprotein (HDL and LDL) of laying hens serum

**Level of PFA** **(g kg** ^-1^ **)**	**Triglyceride** **(mg dL** ^-1^ **)**	**Cholesterol** **(mg dL** ^-1^ **)**	**HDL** **(mg dL** ^-1^ **)**	**LDL** **(mg dL** ^-1^ **)**
**Control**	3343.80	154.75	62.50^c^	29.62^b^
**4**	2973.80	169.13	67.75^c^	41.00^a^
**8**	2830.20	168.00	111.43^b^	48.57^a^
**12**	2667.50	145.25	136.87^a^	45.75^a^
***p*** **-value**	0.49	0.44	<0.0001	0.004
**SEM**	318.15	11.64	3.49	3.11

a,b,c Mean values with different letters in the same column are significantly different (*p *< 0.05).

**Table 7:** Effects of dietary phytogenic feed additive (PFA) levels on ovary weight and number of large and small follicle of ovary

**Level of PFA ** **(g kg** ^-1^ **)**	**Ovary weight** **(g)**	**Large follicle** †	**Small follicle** ‡
**Control**	39.15^b^	4.25	9.25^b^
**4**	44.51^ab^	4.75	18.25^a^
**8**	38.58^b^	4.00	18.00^a^
**12**	49.65^a^	4.25	19.50^a^
***p*** **-value**	0.02	0.38	< 0.0001
**SEM**	1.10	0.39	0.62

a,b Mean values with different letters in the same column are significantly different (*p*<0.05),

† Large follicle: > 10 mm diameter yellow follicle,

‡ Small follicle: 1 to 10 mm diameter small yellow and white follicles.

## Discussion

In the present study, phytogenic additive mixture improved the egg weight only. Similar to this study, Cabuk *et al*. had shown that feed intake, egg production and egg mass did not differ significantly by the essential oil mixture containing fennel seed oil and oregano oil.^6^ In addition, it is reported that only average egg production was increased by polyherbal essential oils in broiler breeder, which contained five herbs (including anise, sage, thyme, fennel seed and oregano).^7^ However, in contrast to current study, egg production was enhanced and FCR was improved by supplementation of essential oils in laying hens.^26^ Ghasemi *et al*. reported no changes in egg production, egg mass and FCR by dietary garlic and thyme in laying hen,^14^ although they found decreased feed intake during six weeks. Khan *et al*. have found no effect on the feed consumption, feed efficiency, egg weight, and egg mass by 0, 20, 60, and 80 g kg^-1^ dietary garlic powder over sixweeks.^27^ Yalcin *et al*. observed that only egg weight was increased by dietary treatments (0, 5, and 10 g kg^-1^ of garlic powder) over a 22-week period.^3^ Improved egg production and egg weight were found by supplementation of 30 g kg^-1^ black cumin in laying hens.^5^

Phytogenic feed additive might improve nutrient digestibility, enhance activities of digestive enzymes and modulate gut microbiota that can caused an increase in broiler performance.

However, different observations by supplementation of herbal feed additive in current study may be due to use of different commercial phytogenic products, level and time of inclusion, and interaction effect of effective compounds of each plant. In addition, levels and interaction between phytochemical constituents of different herbs could have caused the mentioned changes.

In this study, none of the egg quality and yolk parameters were influenced by supplementation of feed additive. In contrast to this study, Zhao *et al*. demonstrated significant improvement in eggshell quality and bone mass after supplementation with daidzein (a phyto-estrogen component).^28^ The estrogens have a variety of functions related to reproduction, including the regulation of calcium metabolism for shell formation.^29^^,^^30^

to fishy egg taint.^16^ In addition to genetics, age of flock and season, gram-positive enteric bacteria play a significant role in the production of TMA especially in the ceca,^18^ because there is higher concentration of TMA in ceca than small intestine.^33^ It is suggested that adding the antibiotics to the diet could reduce the incidence of egg tainting.^18^ Some of the herbs in the phytogenic feed additives of the present study have phytobiotic properties. Garlic is well known for its antibacterial effects.^19^ It is demonstrated that thymol, a major component of thyme, sesquiterpenes in milfoil and anitolin of fennel seed have remarkable antibacterial effects.^20^ Antibacterial effects of herbal feed additive in this study may be responsible of decreased TMA concentration in yolk.

Aromatic compounds of some herb additives such as garlic have potential of unfavorable flavor for consumer, but panelists were not able to distinguish treated eggs from control eggs in aroma, flavor, off-flavor and acceptability. 

Supplementation of polyherbal feed additive caused a decline in TMA of yolk in present experiment and this reaction might decrease unfavorable aroma of egg for above-mentioned panelist. Parpinello *et al. *indicated that rosemary extract did not influence the sensory characteristics when used in substitution to vitamin E antioxidant.^34^ In laying hen, supplementation of 0.5 to 2.0% green tea did not significantly affect these sensory parameters including texture, juiciness, flavor and acceptability.^35^ Hence, further studies are needed to elucidate the effects of phyto-additives on intestinal microflora composition on the egg taste and flavor. Unfortunately, few trials have focused on the effects of herbs on egg sensory quality.

In the present study, the levels of serum HDL in laying hen fed diets containing 8 or 12 g kg^-1 ^herb mixture were increased significantly. Interestingly, LDL levels were increased by supplementation of phytogenic feed additive treatments. However, serum triglyceride and cholesterol concentration were not affected by herb mixture supplementation.

Similar to the present study, Canogullari *et al*. have shown that plasma HDL and LDL levels were higher in birds fed the diets containing 10, 20 or 40 g kg^-1^ garlic powder than those in the control diet.^31^ Azeke and Ekpo have reported the reductions of the total cholesterol, total triglyceride, LDL and HDL observed by garlic powder supplementation at levels of 10 and 20 g kg^-1^ in laying hens diet.^4^ Similar to Canogullari *et al*., Chowdhury *et al*. and Kim *et al*. have shown that plasma LDL-cholesterol was dropped linearly with 5 to 20 g kg^-1^ garlic powder in diet, but HDL-cholesterol concentration was not affected.^31^^,^^32^^,^^36^ Addition of 10 g kg^-1^ thyme in diet significantly decreased plasma total lipid while plasma total cholesterol and LDL were not influenced in commercial and breeding laying hens.^37^ Ghasemi *et al*. have noted that plasma levels of cholesterol, triglyceride, HDL and LDL were not affected by

Similar to the present study, Yalcin *et al*. reported that no significant effects were obtained by supplementation of 5 and 10 g kg^-1^ garlic powder on albumen index, egg shell thickness, and Haugh unit values in layer hen.^3^ In the other study, Yolk index, Haugh unit and eggshell thickness were not affected by dietary treatment, but yolk color was increased by supplementation of 2 g kg^-1^ feed additive.^14^

The egg yolk index and the egg yolk weight were not affected by supplementation of garlic powder. In contrast to the current study, there were significant differences in the egg albumen index, egg shell index, and egg Haugh unit by the 10 and 20 g kg^-1^ garlic powder supplementation.^31^ Egg yolk weights were increased linearly by increasing levels of dietary tamarind in Chowdhury *et al. *study.^32^

In the current study, levels of TMA of yolk were decreased linearly by increase in levels of phytogenic feed additive. Trimethylamine was depressed by supplementing 12 g kg^-1^ herbal additives compared to other experimental treatments. 

Trimethylamine (the highly volatile compound) is more difficult to excrete and can subsequently accumulate in circulation and deposit in developing follicles that leads dietary inclusion of 0.1 and 0.2 g kg^-1^ mixture of garlic and thyme powder.^14^

These conflicting results in relation to mixture of herbs supplementation, level of inclusion and effective component suggested that different effective components of phytogenic feed additive mixture caused these remarkable results in the present study.

Egg production decreases coinciding with weakness in the process of recruitment of follicles into the hierarchy and decrease in ovarian steroids and gonadotropins in old age laying hens.^37^ It is shown that subcutaneous injection of FSH into old hens caused an increase in estrogen concentrations, increased the number of small follicles in their ovaries, and then stimulated rapid growth phase of small follicles in laying hens.^38^

Isoflavones is a major phyto-estrogen that exists widely in natural plants.^39^ Some of the herbs in our feed additive mixture have estrogenic properties. Marigold and fennel seed have flavones and inhibit menopausal symptoms in humans.^40^ Therefore, in the current study weight of ovary and numbers of small follicles were positively influenced by supplementation of herbal feed additive. 

Zhao *et al. *reported that ovary weight or the numbers of hierarchical follicles were not affected by Daidzein (type of isoflavones) supplementation, but oviduct weight was increased.^28^ However, positive effects of phytogenic compound on laying rate and average egg weight were demonstrated during the post-peak stage, but the result of different studies were not comparable because of the differences in phyto-estrogens sources, dose, duration of use and intrinsic oestrogenic state.^41^^,^^42^

In conclusion, dietary supplementation of a phytogenic feed additive (including garlic, marigold, fennel seeds and thyme) could improve egg weight of laying hen. Anti-bacterial properties of this phytogenic feed additive caused reduction of TMA concentration in yolk that was interesting result. Supplementation of phytogenic additive may enhance period of production because of stimulating in small follicle growth. Overall, supplementation 8 or 12 g kg^-1^ phytogenic feed additive resulted in improvement of egg weight and ovarian physiological parameters and reduced yolk TMA concentration.
